# From rat pathophysiology to genomic medicine: an interview with Howard Jacob

**DOI:** 10.1242/dmm.027805

**Published:** 2016-10-01

## Abstract

Howard Jacob is best known for pioneering genomic sequencing of a patient to solve a mysterious pediatric case in 2010. With roots in pharmacology and cardiovascular disease, however, his career has largely been dedicated to dissecting the physiology and genetics of the rat to help understand complex human diseases. Howard was Director of the Human and Molecular Genetics Center at the Medical College of Wisconsin for 16 years, during which time he applied a combination of approaches, including quantitative genetics, integrative physiology and next-generation sequencing, in rat models to shed light on cardiovascular, metabolic and renal disorders. He was a key contributor to the genomic toolbox for rat research, and generated the first targeted-knockout rat models using zinc-finger-nuclease technology. He also contributed to sequencing of the rat genome and establishment of the Rat Genome Database. In this interview, Howard provides his perspectives on the past, present and future of rat-based translational research and explains why, despite his many successes as the leader of a rat group, he recently made the transition to clinical genomics.

## Abstract (precis)

**Summary:** Howard Jacob describes some of the key moments and breakthroughs in his remarkable career, and the path that led him from his research roots in cardiovascular disease and rat physiology to becoming Director of a clinical genomic sequencing program.


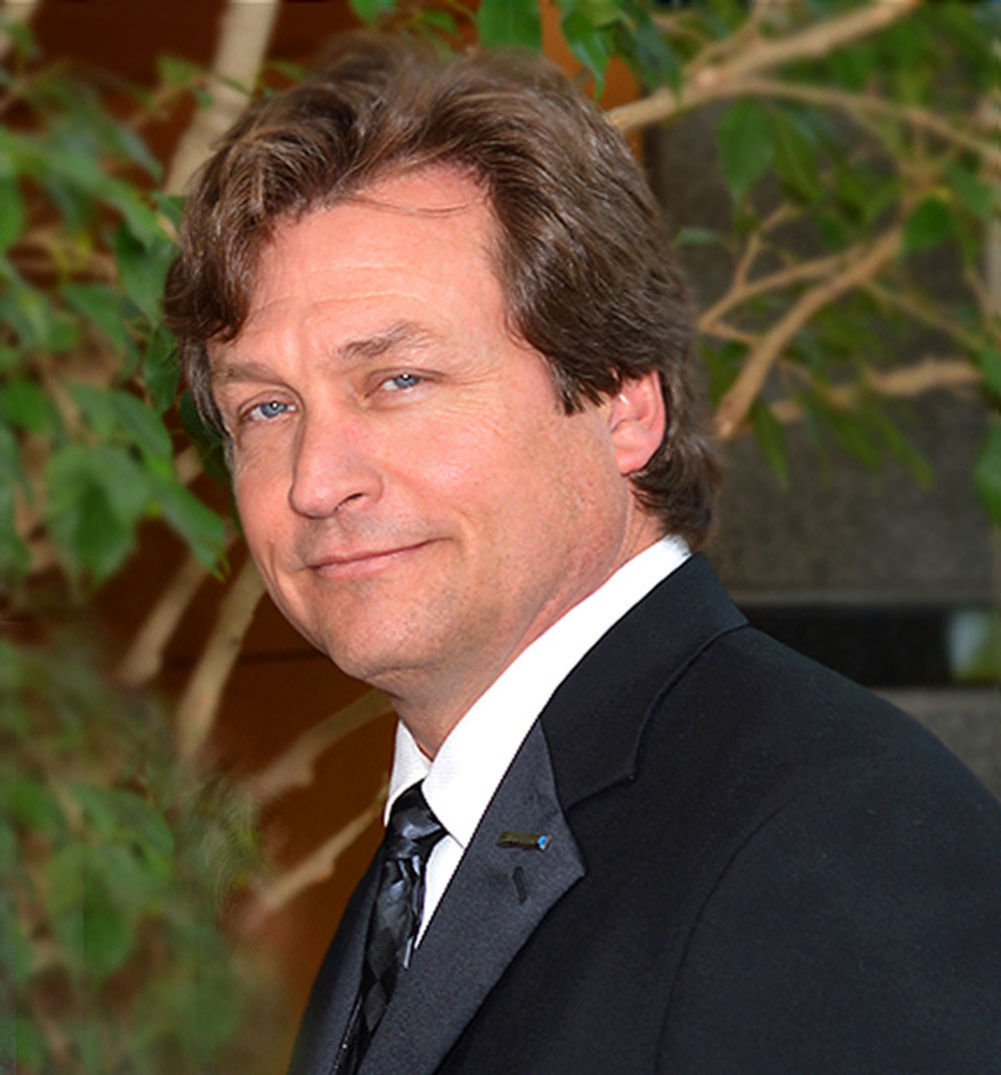


Howard Jacob was born in Kansas, in 1961. He received his BSc in biology from Iowa State University in 1983, and, following a stint as a research technician in Abbot Laboratories, he undertook his PhD in a pharmacology lab headed by Michael Brody at the University of Iowa. He then moved to Harvard and the Massachusetts Institute of Technology (MIT), where he toggled between two different labs – that of Victor Dzau and of Eric Lander – to pursue distinct but complementary projects using mice and rats. In 1992, Howard was appointed as an Assistant Professor at the Massachusetts General Hospital (MGH) and Harvard Medical School. He joined the Medical College of Wisconsin (MCW) in Milwaukee in 1996 and rose in rank from an Associate Professor to Professor in the departments of Physiology and Pediatrics, and, in 1999, became Founding Director of the Human and Molecular Genetics Center. During his years at MCW, he bolstered the institute's genetic and genomic resources and published a series of notable papers that brought the rat model to the forefront of biomedical research. Howard also led the team that sequenced the exome of a pediatric patient, Nicholas (Nic) Volker, who had an undiagnosed gut disease. The venture led to a definitive diagnosis and successful treatment for Nic, representing the first successful use of genomic sequencing in the clinic for an individual patient. The experience prompted Howard to set up the nation's first clinical genomic sequencing program at the Medical College and Children's Hospital of Wisconsin. Since 2015, Howard has been at the HudsonAlpha Institute for Biotechnology in Huntsville, Alabama, where he leads a genomic medicine program that aims to sequence the genomes of 15,000 children with undiagnosed diseases per year.

**Let's start with your early life. Have you always been interested in biology?**

In 1966, when I was 5 years old, we lived on Midway Island in the middle of the Pacific Ocean. My Dad was in the Navy, and there was nothing on this island except a military base. I took to fishing and swimming, and thought the undersea world was incredibly cool. I remember watching *The World of Jacques-Yves Cousteau* on TV and being fascinated. So, from age 5 until my junior year in college, my goal was to get a PhD in Marine Biology. I have been a dork scientist forever.

**What made you change direction?**

The change for me came while working in the early 1980s as a summer employee at Abbott Laboratories. I was working as a Research Technician and starting to think about graduate school. A brother of a friend was a Marine Biologist, so I called and talked to him about it and he warned me that there were not a lot of jobs. Most of the available jobs for marine biologists involved hunting Russian submarines, which I wasn't interested in. I then realized that I enjoyed working for a pharmaceutical company. People are always going to need pharmaceuticals, it's something that can help people, and the work is really interesting. I toyed with the idea of going to medical school, but decided to do a PhD. I was working in Jaroslav Kyncl's cardiovascular lab at Abbott, which is how I ended up in a cardiovascular group in Iowa with Mike Brody – I had become very interested in cardiovascular disease and pharmacology. It is funny that sometimes a position you end up in largely by chance can end up directing a major part of your life.

**What were the major goals of your PhD project and what did you learn from the experience?**

It'll come as no surprise that I worked on rats. My project was focused on understanding how the brain regulates blood pressure. Specifically, we were looking at the mechanisms underlying arterial pressure lability caused by removing the baroreceptors, which play a role in buffering changes in arterial pressure. At that time, everybody thought that the lability after removing the baroreceptors was controlled by the brain, but we found that it wasn't the brain at all. The brain was setting a baseline while local control mechanisms acted as drivers of blood pressure variation. So, although this was a pharmacology lab, we were mostly interested in fundamental pathophysiological mechanisms with relevance to humans.

During this time, I learned that the rat is well suited as a model for human cardiovascular disease, because so much work had been done to characterize the rat's cardiovascular system in both the control and pathobiological settings. Also, the rat has been commonly used by the pharmaceutical industry for decades – I would say that almost every new drug is tested in the rat, to this day. As a pharmacology lab, we knew that if we could understand rat biology better, we could build better models of human disease, which would result in making better drugs.

 “As a pharmacology lab, we knew that if we could understand rat biology better, we could build better models of human disease, which would result in making better drugs.”

**What motivated your move to Harvard?**

In graduate school, I started thinking about what I'd like to do for a job. Back in the days when you didn't have the internet, you had to look at scientific journals to see what jobs were available. Most of the jobs were relevant to molecular biology, which a lot of people using whole animals thought would be a flash in the pan. I thought to myself: ‘everybody is hiring molecular biologists, but somebody has got to figure out how you link the molecular mechanisms to the physiology of the whole animal’. If you take as an example a new drug, if it doesn't work in the whole animal, it doesn't matter what it does in the cell.

This drove me to look for a post-doc position in a molecular biology lab. I ended up working with Victor Dzau, who was at Brigham and Women's Hospital at Harvard at that time [1989]. Victor used transgenic mice rather than rats, but he had wanted me to join the lab because I had experience in studying physiology in rodents. I was working on ways to measure blood pressure in a mouse, which was technically really challenging because a mouse is teeny compared to a rat. I was the first to use a catheter to measure blood pressure in a freely moving mouse. While working with transgenic mice, Victor introduced me to Eric Lander at MIT. Eric is a smart guy – he is only a few years older than me and he was already an Associate Professor at Harvard Business School and a Fellow at the Whitehead Institute. He had an idea to use genetic markers to make a genetic map of the rat genome, which in turn could be used to map the genes responsible for common complex diseases using quantitative genetics. He and Victor started working together on the Rat Genome Project – long before Eric started on the mouse – and I was the lead post-doc on that project. I was now doing two post-doc projects in parallel: mouse physiology on transgenics with Victor, and rat molecular genetics with Eric. I don't recommend doing this; it's not the smartest move in terms of lifestyle, but it was fruitful. At the time, the information we had about the rat genome was limited. This project represented a great opportunity to learn more about the genetics of the model. It also helped me to learn more about rat biology. What I found was that genetics was the link between cellular biology and whole-animal physiology, just what I had hoped to do.

**Did the lack of information about rat genomics contribute to making the mouse the dominant model in biomedical research?**

Absolutely. The challenge with rats has always been that everybody has interest in the biology of the model, but the genetic/genomic tools lagged far behind the mouse, which was commonly used for genetic studies. When I started out working on genomic tools for the rat, I didn't realize how hard it was going to be. The field was small – I've always tried to stay away from overcrowded areas of research – and I hadn't realized it would take me 20 years to get the genomic toolbox pulled together. I kept at it because I always believed that the rate-limiting step would be characterizing the animal's physiology, and we had already made headway in the rat. In fact, it has taken mouse researchers 20+ years to get the basic physiology out there. In a sense, mouse research was pulling in one direction and rat research in another, but it all worked out. It was an interesting time. I had a sense of purpose in building reagents that other people could use, and thus would benefit the field as a whole. This also gave me the opportunity to collaborate with a lot of really smart people.

**What made you decide to move over to Wisconsin, after four fruitful years at Harvard?**

There were two main reasons. First, there was so much genomics going on in Boston and so many genomicists that I felt I would struggle to distinguish myself. The second reason was that we had a 2-year-old and our families were out in the midwest. I started job hunting and I got two very different offers. One was to go to Huntsville Alabama and work for a company called Research Genetics and the second was to set up a lab at Milwaukee. I originally accepted the offer to go into industry but, after 2 weeks, I decided I really wanted to have my kids raised closer to my family. I was impressed by MCW because they had one of the best rat physiology departments in the world. With the rat genomic tools that we now had and an amazing physiology group, I knew I was in the right place to be able to do cutting-edge research and make an impact.

**In the 19 years you were at MCW, what would you say were your greatest achievements?**

There was virtually no genetics footprint at the institute when I first got there in 1996, and I ended up becoming the Founding Director of the Human and Molecular Genetics Center in 1999. We went from having two faculty members in the center to 30 by the time I left. Genetics and genomics are all over the campus now, so I would say that this is my biggest legacy.

The other contribution I feel good about is building up the infrastructure for rat research. Techniques and tools that are now used commonly by rat researchers – sequencing, databases and resources for sharing data and models – plus an understanding of the genetic underpinnings of basic physiology and pathophysiology of the rat, are all out there now, and didn't exist before. I'm proud of working with my colleagues there at MCW to build tools and establish the functional genomics of the rat. Before leaving, I passed on all of my rat projects to my colleagues at MCW, except one that I have been working on with Eric since being a post-doc in his lab. We finally have the tools to be able to address long-unanswered questions about hypertension-induced renal disease in the Fawn-Hooded rat, and I intend to complete this project myself.

**You were also involved in setting up the early rat-focused meetings. What impact do you think these meetings had and will continue to have in coming years?**

The idea for a rat meeting first came from Douglas Vollrath, who had been a post-doc at the Whitehead Institute in David Page's lab when I was in Eric's lab. Doug, who is interested in understanding the genetics of eye disease, had recently shifted from using mice to rats, and we felt that we needed a meeting to help build the tool base. Now that the toolbox is more developed, I still think it is important to have the rat meetings for people to get together and share stories and knowledge: to meet others in the field. These interactions are vital for the exchange of information and to improve science. But I think the community now has to decide what they want to do with these interactions. Do they want to do large-scale projects together? The glue that held the community together when I was involved was our shared goals: to build tools, build reagents and get the rat recognized. We have kind of done that, and the community now needs to figure out what next steps would continue to drive intellectually rich scientific meetings that are rat-centric.

It's also important to note that the world is changing. I've completely changed my research focus so that I now look at genetic variation in the human and try to work out what biological mechanisms are affected by this variation in the rat, instead of trying to figure out how a gene functions in the rat and then extrapolating this information to the human. With advances in gene editing and so forth, we can engineer rats in whatever way we want, so to me it makes sense to take information from a patient and then test its biological impact in the rat. I think that this is where the field needs to move – how do you make the rat a better test-tube for clinical studies?

 “With advances in gene editing and so forth, we can engineer rats in whatever way we want, so to me it makes sense to take information from a patient and then test its biological impact in the rat.”

**Was Nicholas Volker's case your first foray into genomic medicine? What was the story behind this, and what impact did it have?**

What most people don't know is that when I became Director of the Human and Molecular Genetics Center, I wrote a mission statement. The mission statement was to enable genomic sequencing, to advance research and ultimately to improve patient care. In 2002, Francis Collins [National Institutes of Health (NIH )Director] called me to talk about sequencing the rat genome. I, and others, had put a lot of effort into convincing the NIH that this was important, and it had paid off. Whereas it took 10 years and a billion dollars to sequence the first human genome, the rat genome was completed by 2004, and the cost had gone down to ∼100 million dollars. In 2004, I stood up in front of the executive faculty at MCW and said we are going to have genomic sequencing in the clinic by 2014 – everybody laughed at me. I estimated, given the dramatic improvement in costs and speed that had happened already, that by 2014 the technology would be cheap and accessible enough to be able to use clinically. From that time I started hiring faculty members and developing the genomics programs at MCW to accomplish this goal. I knew we were never going to be a genome-sequencing center – that ship had sailed – but I wanted to be the first to implement sequencing for clinical care. I didn't know what that would entail exactly; however, I believed that building the infrastructure to tackle complex phenotypes in rats, along with molecular genetics and genomic sequencing, would help position the group for medical genomics.

At the end of June 2009, Alan Mayer sent me an email. Alan was a Gastrointestinal Pediatrician at the Children's Hospital of Wisconsin, and I had known him peripherally when I was at MGH. He asked if we could sequence Nic's genome to figure out what was wrong with him, as he was dying and they didn't know why. There are details about the case in a book that was recently published: *One in a Billion: The Story of Nic Volker and the Dawn of Genomic Medicine*, which was written by Pulitzer-Prize-winning journalists. I met with Mike Tschannen, who was a Technician in my lab, and Liz Worthey ,who was then a Research Scientist with the Rat Genome Database, and asked them if we could do it. They said yes. We embarked on the mission at an interesting time [2009] – the economy had tanked and there was no money to fund this. I had a little rat biotechnology company called PhysioGenix, and I met with the Board after a meeting and told them Nic's story. I said that we wanted to do clinical medicine and now had the opportunity to start with a pilot case. Ultimately, the Board helped pay for it, along with the Roche company 454 Life Sciences. It took us 6 weeks to do the sequencing and it took us 6 months to analyze the data. We ended up finding out what was wrong with him and then there was the question of what we could do about it. The solution was to do a cord-blood transplant for a previously unknown gut disease. Nic is still alive today and is doing very well – his gut disease appears to be under control.

Later, I met Nic and his family, and the entire experience changed my life. I had always believed that genomic sequencing could change medicine and I now knew it could. My passion has become to get sequencing to be routine in the clinic. This is basically why I left the rat field. The science we were doing at MCW was the coolest stuff I have ever done, but I'm 55 now and realize that I don't have a lot of time left in my career. I want to finish my career by helping people get access to genomic sequencing and to use animal models to help provide biological answers that will help them. Towards this goal, I recently started collaborating with the Children's Hospital of Alabama on a pilot project dedicated to diagnosing 100 sick kids. I'm at the point where I want to do these ventures on a bigger scale.

 “I had always believed that genomic sequencing could change medicine and I now knew it could. My passion has become to get sequencing to be routine in the clinic.”

**Exome sequencing versus whole-genome sequencing: which is ultimately going to be more powerful in the clinic?**

In my mind, there's a simple answer to this. ENCODE [Encyclopedia of DNA Elements] showed that 80% of the genome is biochemically functional. Most of the clinical laboratories are using exome sequencing and are making a diagnosis 25-30% of the time. If you only look at 1.5% of genome and aren't able to make a diagnosis the majority of the time, it's obvious that the rest of the genome is important. Many leaders in genomics who initially pushed to do whole-genome sequencing are now saying let's just look at the exome for the sake of power versus price. I don't get it: we can't learn about the rest of the genome if we don't look at it. We have seen this movie before, first SNPs [single-nucleotide polymorphisms], then exome, then whole genome. It's obvious that the exome alone is not enough. Of course it's more expensive to sequence the whole genome, but leaving critical knowledge behind is worse and is just kicking the can down the road. Critical information to solve key clinical problems lies in the whole genome. To the investigators and physicians out there doing exome sequencing, I ask what they would do if the patient involved was a family member or friend. I would be shocked if any of them said, “I'm just going to analyze 1.5% of the genome”. What the exome guys are doing reminds me of the old adage about the person looking for his keys under the streetlight – he keeps looking although they're clearly not there, because it's the only place he can see. It's something I feel passionately about, especially now that we can sequence and analyze the genome for a clinical case in 30 days for $6500. It takes $12,000 to make a knockout rat, and here we're talking about the chance to make an impact on a patient's life.

**What role will animal models play in this era of genomic medicine?**

There is definitely a place for model systems in this world. There are a lot of data being generated in clinical sequencing labs. We have pinpointed a number of interesting variants from patients, and models such as the rat are ideal to explore their functional implications. There is a lot of work that needs to be done, and it's important to note that the end goal isn't to just answer intellectual questions. There are patients involved and we want to gain an understanding that will ultimately modify their care, if possible. That's a really powerful driver for me – much more than getting a paper. With all the data that is being generated through next-generation technologies, models are going to be crucial for translation of basic findings that set the foundation for new medical therapies.

 “With all the data that is being generated through next-generation technologies, models are going to be crucial for translation of basic findings that set the foundation for new medical therapies.”

I think that the future of rat-based translational research is incredibly bright. The complex physiology of the rat – which mirrors that of humans for many traits – offers the ability to delve deep into the underlying pathways of multigenic diseases, such as hypertension and diabetes. The rat provides a great model to explore the individual gene variants we're finding through human genetic studies, and to assemble the complexity we see in common human diseases. The question is important, however, of whether other models might be better depending on the aspect of biology under investigation. Using behavior as an example, the rat is a better behavioral model than the mouse, but my understanding is that the mouse is a better model in studies of the immune system. People have to do their homework: why are you picking the model, how close is its biology to that of humans and what is available in the toolset?

 “The complex physiology of the rat – which mirrors that of humans for many traits – offers the ability to delve deep into the underlying pathways of multigenic diseases, such as hypertension and diabetes.”

I often hear people say that “rats don't do *x*, and so they're not a good model for this”. Usually, the conclusion is based on studies of only one rat strain – people don't always look at the entire collection of rat strains. There is a lot of ignorance and a lack of understanding that a single strain of rat cannot capture the diversity within the species. The Rat Genome Database has been dedicated to building and cataloguing an amazing amount of data across organisms for years. I encourage people to ask questions using this database and by probing the literature first, and to then design your study based on a deep understanding of animal biology. There are so many things we can do with rats now, especially with the gene-editing toolset, and I think we're going to see some really cool science.

**Whether outbred or inbred models are best for disease studies has been widely debated. What is your opinion?**

I've been involved in this debate for 30 years. There is a really simple answer that not everybody wants to hear. Outbred strains are better for questions where genetic diversity is important and you need a large number of animals. Human clinical trials involve 10,000 people. Why? Because they are outbred. It's not economical to try to perform a physiology test on tens of thousands of animals derived from outbred strains. For optimum reproducibility, you have to use inbred animals, so that they are as genetically similar as possible to each other. An inbred animal is the same as studying one patient, which you wouldn't do – the individual cannot mimic all of humanity. The scientist has to think about the question they are asking, and exploit the many tools we now have to streamline the study design. Genomic sequencing can be used to determine which animals are more or less related, and with CRISPR/Cas9 and other gene-editing tools you can make specific mutants and ask what the same mutation does in different genetic backgrounds. Nowadays, you can be quite clever in the way you design experiments using animals. At my old company [PhysioGenix], we took four inbred rat strains and combinatorially bred them to obtain six different F1 hybrids. These hybrid strains are identical individually yet collectively captured 80% of the variation in the rat genome known at the time. This approach can be used to gain insight into whether the genetic background impacts the response of a strain to a particular drug, for example. So, you can leverage inbred strains to create known genetic heterogeneity and control for it by making F1 hybrids. There has been an ongoing battle on whether inbred strains or outbred strains are better and I don't expect this issue to go away soon.

**So, the future of rat research looks bright, but what are the key challenges that the community needs to overcome?**

The biggest challenge is to not get trapped in a fight between investigators in the different fields as to which of the different model organisms is best. I think it's important to always start by knowing what clinical question you are addressing – whether it is to test a therapy, determine a mechanism of action, or explore the biology of a disease – and to then ask which model system is going to be the most appropriate. The trick is to make sure your science is important. I have walked between genomics, physiology and molecular biology but stayed focused on questions that I felt were important and that my team could answer. I didn't chase things that we weren't any good at. I'm not saying we didn't try some crazy experiments and that things didn't fail, but for the most part we tried to learn from other model systems, bring existing technologies to the rat, and to leverage the strengths of the rat physiology. It's an amazing model system, and the onus is on investigators to deploy it in a way that answers important questions.

 “…it's important to always start by knowing what clinical question you are addressing…and to then ask which model system is going to be the most appropriate. The trick is to make sure your science is important.”

**You had a number of great mentors during your career. Who has had the biggest influence on your style of running a lab?**

I was incredibly fortunate to be trained by three really busy and successful guys and I learned a lot from them. With Victor and Eric, I had the advantage of being able to watch and learn from two people who came from entirely different backgrounds. Victor was a clinician who was running a molecular biology group from the perspective of a clinician, while Eric, who has a DSc in Mathematics from Oxford, was pioneering whole-genome mapping for common complex diseases in novel ways. And then there was Mike Brody. Mike was very difficult to work for as a new graduate student because he basically threw you in at the deep end. On the first day, he would hand you a ∼500-page program project grant and say, “read this and tell me what you want to do”. Having worked at Abbott Labs, I knew what an open project was and what a closed project was, and knew that my project had to have an end. Whether it works or doesn't work, you've got to have an end, otherwise it goes on forever. The project I fixed upon involved a technique that nobody else in the lab was using, so I needed to figure out how to do it – it took me months and months before I could get anything to work. I was very frustrated because we had a lab meeting only around four times a year and Mike would schedule it the day before, and ask me to bring my raw data. Then I'd be up all night analyzing the data I should have analyzed weeks ago. Mike only made time for senior students who had gotten past the technical hurdles and had interesting data. I really didn't like it at the time, but when I got to be a post-doc I realized that the experience taught me how to solve problems, build collaborations, seek solutions, and not wait for somebody to tell me what to do and how to do it. So, while difficult, it made me a better scientist, and I use a similar strategy with my students, although we have lab meeting weekly.

Those three individuals – Mike, Victor and Eric helped to shape everything that I do now. So I don't tell my graduate students what their project will be – they've got to figure it out for themselves. I discovered that choosing your own project and solving problems independently is the key to becoming a successful scientist. My job is to help guide my lab members but not to tell them step-by-step what to do.

 “I discovered that choosing your own project and solving problems independently is the key to becoming a successful scientist.”

**What other advice do you offer to early-stage investigators?**

By the time you are done with your post-doc, everybody is as smart as you are, and everybody works hard. So, how do you distinguish yourself amongst a ton of hard-working, smart people? What makes you unique? My key advice is to not be afraid to tackle a hard question and to go down a different route. If there are 1000 people doing the same thing you are doing, the chances of you generating something of significance is low. Second, you've got to know how to work with other smart people. The days of being a lone wolf in science are over. You need to make a contribution as part of a talented pack. Sometimes that means you're working harder than you might like or doing things that you think are a waste of time, but the insights you get from working with a smart pack can open up new avenues for your research. I've had a lot of smart post-docs and students who wanted to take the lab in directions that I didn't initially agree with, but I usually let them run a little bit and often it led to interesting questions. It is important to be open-minded, and to not be afraid of failing.

I'm also trying to get more and more young, smart people to get involved with big data analysis – we need more people with computer science expertise in biology. Everybody talks about the big data challenges, but we haven't even got started yet; we haven't even scratched the surface. Humans are the real big data problem. We each have 100 trillion cells. If you took all the DNA in an individual human's cells and stretched it out, it would reach the sun and back 666.5 times. If we knew the blueprint for every cell, if we knew how cells worked together, I think we'd be in able to practice better medicine. We need smart people to get involved in thinking about data on a different scale and in putting together that blueprint.

**If you hadn't chosen science, what would you be doing now?**

I don't think I would do anything else. Since the age of 5, there's never been anything else that I wanted to do. When I finish this next gig, I'd like to teach high-school science. I think getting kids excited about science is really important, and now that I can teach from the perspective of being a scientist, hopefully I can do a good job. Most people who have made a career in science knew what they wanted to do early on, and can usually name that one teacher who got them excited about science. That's my retirement plan – it would kill me to put me on a beach with nothing to do!

**What do you enjoy doing out of the lab?**

It won't surprise you that I'm adventure-driven. I enjoy scuba diving and skiing with my family, and also do triathlons. I thrive on adrenalin rushes. I've also jumped out of aeroplanes. I also love food, and to read books on history, some classics as well as fictional novels. I wish I could say I had a hidden talent like some colleagues who are wonderful musicians or creative in other areas – I don't have that, but I do need to be doing *something*.

